# Mechano-Filtering Encapsulation: A Stitching-Based Packaging Strategy Implementing Active Noise Suppression in Piezoresistive Pressure Sensors

**DOI:** 10.3390/mi16040486

**Published:** 2025-04-20

**Authors:** Yi Yu, Yingying Zhao, Tao Xue, Xinyi Wang, Qiang Zou

**Affiliations:** 1 School of Microelectronics, Tianjin University, Tianjin 300072, China; yu2417506114@163.com; 2 Tianjin Flying Pigeon Group Co., Ltd., Tianjin 301600, China; 18622912991@163.com (Y.Z.); 13116091576@163.com (X.W.); 3 Center of Analysis and Testing Facilities, Tianjin University, Tianjin 300072, China; xuetao@tju.edu.cn; 4 Tianjin International Joint Research Center for Internet of Things, Tianjin 300072, China; 5 Tianjin Key Laboratory of Imaging and Sensing Microelectronic Technology, Tianjin University, Tianjin 300072, China; 6 State Key Laboratory of Advanced Materials for Intelligent Sensing, Tianjin University, Tianjin 300072, China

**Keywords:** porous sponge, piezoresistive sensor, noise suppression, pre-stressed stitching encapsulation

## Abstract

Flexible pressure sensors face the dual challenges of weak signal extraction and environmental noise suppression in wearable electronics and human-machine interfaces. This research proposes an intelligent pressure sensor utilizing chitosan/carbon nanotube/melamine sponge (CS/CNT/MS) composites, achieving high-performance sensing through a dual-stage noise reduction architecture that combines mechanical pre-filtration and electrical synergistic regulation. An innovative compressed-stitching encapsulation technique creates pressure sensors with equivalent mechanical low-pass filtering characteristics, actively eliminating interference signals below 3 kPa while maintaining linear response within the 3–20 kPa effective loading range (sensitivity: 0.053 kPa^−1^). The synergistic effects of CS molecular cross-linking and CNTs’ three-dimensional conductive network endow the device with a 72 ms response time, 24 ms recovery speed, and over 3500-cycle compression stability. Successful applications in smart sport monitoring and tactile interactive interfaces demonstrate a material-structure-circuit co-design paradigm for mechanical perception in complex environments.

## 1. Introduction

Flexible pressure sensors have attracted significant attention as emerging sensing devices in recent years. Their exceptional wearability [1,2,3,4,5], biocompatibility [6,7], and adaptability to multi-scenario applications [8,9] demonstrate unique value across fields including human motion monitoring, human-machine interfaces, and bioinspired electronic skin [10,11,12,13,14]. Among prevalent sensor types (piezoresistive [15,16,17,18], capacitive [19,20,21], and piezoelectric [22,23,24]), piezoresistive pressure sensors stand out due to their high sensitivity (typically reaching several kPa^−1^ level), rapid response (<100 ms), and cost-effective manufacturing, establishing them as a focal point in flexible sensing research.

Currently, the performance enhancement of piezoresistive sensors primarily relies on the microstructure design of sensitive layers and optimization of conductive networks. Researchers have significantly improved sensor sensitivity and pressure response ranges by introducing carbon-based conductive materials (e.g., carbon nanotubes (CNTs) [25] with rigid high conductivity, carbon black (CB) [18,26] with plastic deformation advantages, graphene [27,28] with large specific surface area effects, and MXene [29,30,31,32] with metal-level conductivity) and constructing various structural designs (including wrinkled geometric configurations [33], spike-like microstructures [34], three-dimensional porous architectures [35,36,37], and biomimetic structures [38,39,40,41]). However, these approaches often face common issues of insufficient linearity and weak anti-low-frequency interference capabilities, limiting their reliable applications in high-noise environments.

This study proposes a novel flexible pressure sensor through synergistic design of composite materials and mechanical encapsulation. Using melamine sponge with >99% porosity as the 3D porous scaffold, the chitosan/carbon nanotube (CS/CNT) composite sensing layer was constructed via progressive impregnation. The synergistic effect between chitosan’s bioadhesion and CNTs’ conductive pathways ensures exceptional stability over 3500 compression cycles. Compared to Wang et al. [42]’s TPU/PU-based sensor with segmented response (0.1 kPa^−1^ at 0–8 kPa vs. 0.01 kPa^−1^ at 8–23.3 kPa), our dual−mode noise suppression architecture achieves full linear response in 3–20 kPa: (1) mechanical low-pass filtering through compression-stitched encapsulation effectively attenuates environmental vibrations; (2) CNT network self-activation above 3 kPa autonomously blocks random contact noise. This cross-scale design delivers stable sensitivity (0.053 kPa^−1^) and rapid response/recovery (72/24 ms), providing a reliable solution for continuous pressure monitoring applications.

## 2. Materials and Methods

### 2.1. Materials and Chemicals

Chitosan was purchased from Sinopharm Chemical Reagent Co., Ltd. (Anhui, China) Multi-walled carbon nanotubes (CNTs; length: 5–15 µm; diameter: 10–30 nm) and dispersants were sourced from Chengdu (China) Jiacai Technology Co., Ltd. (Chengdu, China) Melamine sponge (porosity > 99%) was procured from Shanghai (China) Zhenmo New Material Co., Ltd. (Shanghai, China). All chemicals were used as received without further purification. The sewing thread used was a 402 high-speed polyester sewing thread.

### 2.2. Preparation of CS/CNT Solution

First, 0.56 mL of acetic acid (CH_3_COOH; purity 99.5%) was added to 100 mL of deionized water. Then, 1.0 g of chitosan powder (deacetylation degree 80–95%) was gradually introduced into the acetic acid solution and stirred until complete dissolution to obtain a homogeneous, slightly viscous CS solution. According to the CNT/CS mass ratio of 1:1, 0.5 g of multi-walled carbon nanotubes and 0.5 g of aqueous dispersant were sequentially added to the CS solution. The initial surface wetting and uniform distribution of CNT particles were achieved through stirring. Ultrasonic dispersion was performed using an ultrasonic homogenizer, with 3-min sonication followed by 3 min settling for cooling, repeated for 1 h. Finally, the solution was filtered through a 300-mesh sieve to obtain the CS/CNT solution.

### 2.3. Fabrication of Sewing-Based Encapsulated CS/CNT Sponge Sensor

**Figure 1:**
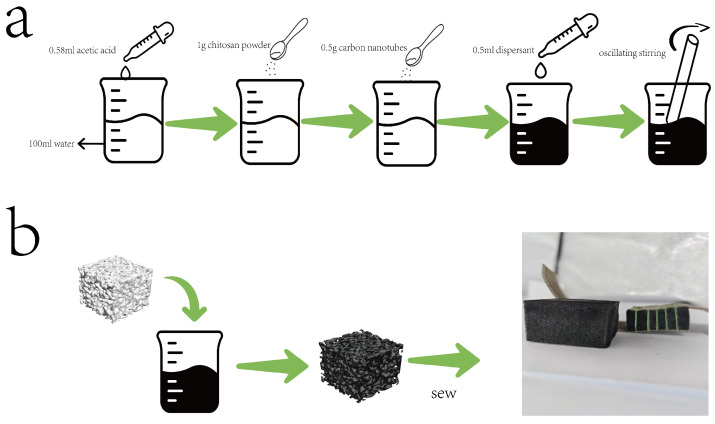
Sensor preparation process, (**a**) solution configuration, (**b**) preparation of composite sponges by immersion drying.

A commercial melamine sponge (30 mm × 30 mm × 20 mm; porosity > 99%) was selected as the flexible skeleton. The internal microdust was removed through alternating ethanol−deionized water rinsing (3 times), and the sponge was pretreated in a 60 °C blast-drying oven for 2 h to ensure dryness. The cleaned sponge was fully immersed in the previously prepared CS/CNT solution and subjected to three consecutive impregnation cycles to ensure the complete infiltration of the solution into the micropore structure. The saturated sponge was then initially cured at 40 °C for 2 h to prevent structural collapse caused by rapid dehydration, followed by drying in an 80 °C drying oven for 8 h, ultimately obtaining the black conductive CS/CNT/MS composite sponge. The overall preparation process is shown in Figure 1.

A 0.5 mm precision punching mold was used to fabricate a 5 × 5 array of holes (spacing: 5 mm; depth: penetrating the substrate) on the dry conductive fabric surface, forming a periodic stress-guiding structure. The sensor structure consisted of a five-layer configuration, with the central layer being a CS/CNT/MS, while the upper and lower sections each comprised two stacked layers of conductive sponge and conductive nonwoven fabric, respectively, adhered to the top and bottom surfaces of the central CS/CNT sponge layer.

All encapsulation used 402 high-speed polyester sewing thread (high-strength polyester fiber material, with a diameter of 0.3 mm, tensile strength > 120 N, and elongation < 3%), which ensured teh structural stability of the sponge substrate during dynamic compression due to its minimal deformation and excellent abrasion resistance. Thread tension was controlled via pre-compression fixation: the sponge substrate was pre-compressed to a thickness of 15 mm using a custom-designed mold before sewing operations, ensuring the uniform embedding of stitches into the material’s porous structure and preventing encapsulation loosening. Standardized stitching requires double-thread sewing with stitch spacing strictly controlled at 5.0 ± 0.1 mm (calibrated according to the aperture parameters of the conductive nonwoven fabric) and an S-shaped reciprocating stitching path implemented along the longitudinal edge of the sponge substrate.

The study prepared three parallel sensor sample groups (each containing three independent sensors) for each test group. All critical performance tests were repeated with at least three independent samples to ensure the statistical reliability of the data.

### 2.4. Characterization and Measurements

A scanning electron microscope (SEM, Regulus 8100, Hitachi, Beijing, China), optical microscope (Oplenic Digital Camera Oplenic, Hangzhou, China) were used to capture the surface morphology of the pressure sensor. Pressure was applied using a force gauge (AIGU-ZP-1000, AIGU, Shenzhen, China) in combination with a self-assembled, high-precision displacement meter setup, with testing conducted at room temperature.Changes in sensor resistance were measured using an LCR meter (Keithley 2110, Tektronix, Shanghai, China). A DC power supply (KXN-305D, ZHAOXIN, Shenzhen, China) provided a 3V voltage to the LED bulb. An illuminance meter (DL333204) was used to quantify the luminous intensity of the LED lights.

## 3. Results

### 3.1. Physical Structure Characterization

**Figure 2:**
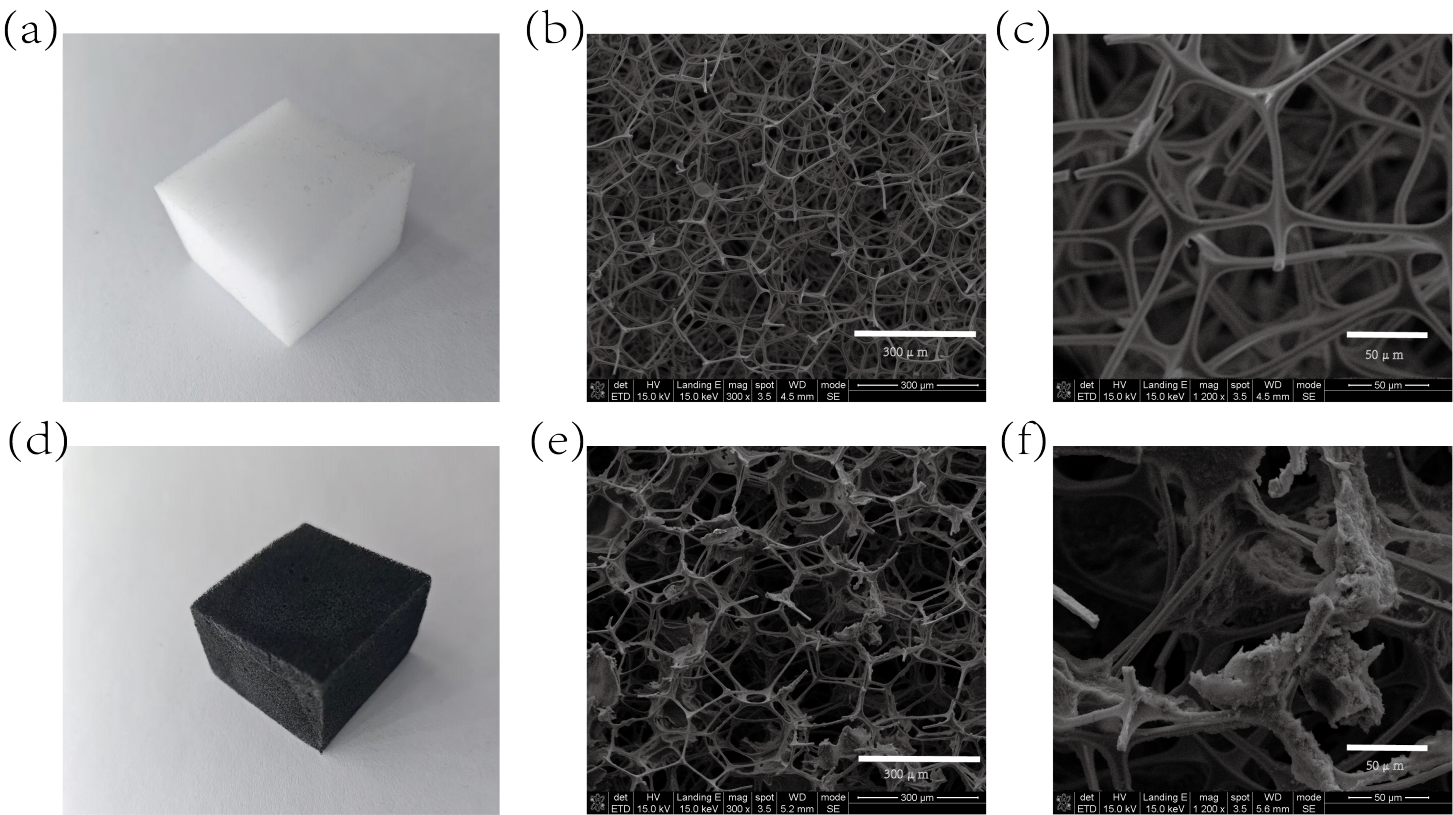
Sensor characterization. (**a**–**c**) SEM images of MS at different scales; (**d**–**f**) SEM images of CNT/CS/MS at different scales.

To achieve efficient and stable conductive network construction, this study applied directional chemical adsorption to load the CS/CNT composite conductive material onto the porous skeletal surface of the melamine sponge (MS). Macroscopically, the impregnation process transformed the original off-white color of the MS matrix into a uniformly deep black color, visually confirming complete coverage by conductive components. Figure 2 presents SEM micrographs showing the following: The original MS exhibited typical dodecahedral open-cell structures with smooth skeletal surfaces. In the CS/CNT/MS samples post-impregnation, the MS skeletal surfaces formed CS adhesive layers with attached CNTs, establishing a three-dimensional interconnected conductive network, further confirming the attachment of CS/CNTs to the sponge skeleton.

### 3.2. Sensing Mechanism

**Figure 3:**
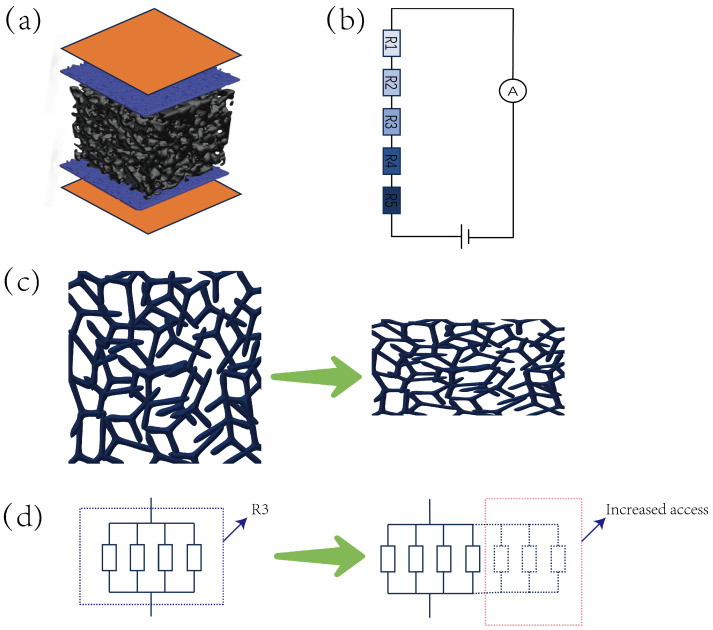
The operational structure of the CNT/CS/MS pressure sensor: (**a**,**b**) The sensor’s resistance model and equivalent circuit. (**c**) The deformed sponge skeleton under applied pressure. (**d**) A schematic of increased conductive pathways.

As illustrated in Figure 3a, the working mechanism of this sensor is based on a five-layer composite conductive pathway model, where the total resistance can be expressed as

(1) $$R_{total}=R_{1}+R_{2}+R_{3}+R_{4}+R_{5}.$$

In this configuration, $R_{1}$ and $R_{5}$ represent the resistances of conductive nonwoven fabric layers, $R_{2}$ and $R_{4}$ denote conductive sponge resistances, while $R_{3}$ corresponds to the resistance of CNT/CS/MS composite sponge. The static electrode properties of conductive nonwoven fabric layers ($R_{1}$, R_5_) (30 × 30 mm^2^; sheet resistance <0.05 Ω; thickness tolerance ±1 µm) combined with low-impedance conductive sponge layers (R_2_, R_4_; measured impedance 1.0 ± 2.0 in 1 mm-thickness regions) demonstrate <0.1 % impedance fluctuation within the 0−20 kPa operational range, allowing simplification as constant resistances. The core sensing unit (CNT/CS/MS, R_3_) follows a hierarchical contact network model: in a relaxed state (Figure 3c), limited conductive path combinations spontaneously form within the skeletal framework; axial pressure induces elastic buckling deformation; and the R_3_ microstructure inherently contains conductive pathways under zero pressure, forming the sensor’s baseline resistance. As shown in Figure 3d, incremental pressure reduces inter-skeletal spacing and creates new conductive paths (denoted by dashed lines and red boxes). These additional resistances exhibit parallel connectivity with existing paths, leading to rapid total resistance decrease as pressure increases.

**Figure 4:**
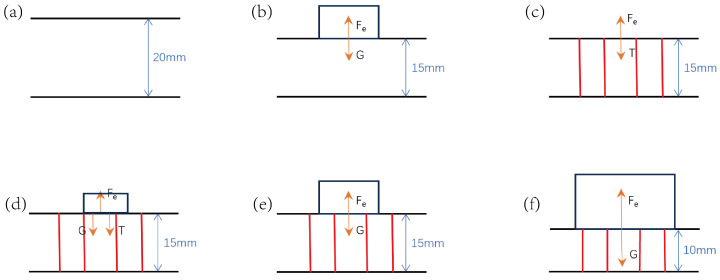
Principle of sensor compression operation: (**a**) Initial 20 mm-thick sponge. (**b**) Placing object a to compress sponge by 5 mm. (**c**) Force analysis when sponge is fixed at 15 mm through sewing. (**d**–**f**) Force analysis when placing objects b, c, and d, respectively.

As shown in Figure 4, when a load is applied to the original sponge, it produces deformation. When object a is placed such that the sponge experiences exactly 5 mm of compressive deformation, the load provided by object a is approximately 4.138 N (Figure 4b). Implementing prestress-locking encapsulation in this stage results in an intrinsic prestress equilibrium system within the sensor under zero external loading conditions.

(2) $$F_{elastic}=F_{textile}+F_{external}.$$

The equation terms are the following: $F_{elastic}$ is the elastic restoring force of the sponge skeleton, $F_{textile}$ denotes sewing thread tension, and $F_{external}$ represents the applied external load. When object b (Figure 4d) is placed and the external load $F_{external}$ < $F_{th}$ (threshold force), the system maintains constant deformation (Δh=0) via thread tension adjustment, resulting in nearly unchanged sensor impedance output. When object c (with mass identical to object a) is placed and $F_{external}$ = $F_{th}$ (Figure 4e), the thread reaches a critical state where the tension $F_{textile}$ = 0. When object d (with mass exceeding a) is placed and $F_{external}$ > $F_{th}$ (Figure 4f), the thread slackens ($F_{textile}$→0), allowing the sponge to undergo secondary deformation, which alters the resistance. This mechanism enables mechanical signal filtering in low-pressure ranges while maintaining high sensitivity in high-pressure regimes.

### 3.3. Basic Sensing Characteristics

A series of tests were conducted to demonstrate the characteristics of the CS/CNT/MS pressure sensor. Figure 4a,b illustrates the schematics of the MS before/after the impregnation and sewing process. As shown in Figure 4c–e, we measured the variation in the relative resistance of the 20 mm-thick and compressed 15 mm-thick MS under applied pressure, evaluated across different concentration conditions. By comparing Figure 4c,d, a significant variation in sensor sensitivity is clearly observed. The sensitivity of the sensor is defined as follows:

(3) $$S_{p}=\frac{\Delta R/R}{\Delta P}kPa^{-1}.$$

where ΔR is the resistance change caused by pressure, R is the initial resistance, and ΔP is the applied pressure. For the tests of the 20 mm uncompressed sensor, the curve shows a large slope before approximately 4 N, demonstrating excellent sensitivity. At a 4 kPa pressure, sensors with different concentrations exhibited nearly identical ΔR/R values. A possible explanation is that for sponges of different concentrations, although their initial resistances differed slightly, under identical pressure, the increase in conductive pathways might have been similar, resulting in comparable resistance change rates. As pressure increased further, the sponge became nearly flattened, causing the conductive skeleton to make full contact and drastically reducing resistance. Thus, the ΔR/R of all concentrations approached 1. The high sensitivity in 20 mm sensors without pressure may have arisen because, at 0 pressure, even high-concentration sponges had few conductive pathways. Slight pressure application potentially doubled the conductive pathways, reducing resistance to 50% of R.

**Figure 5:**
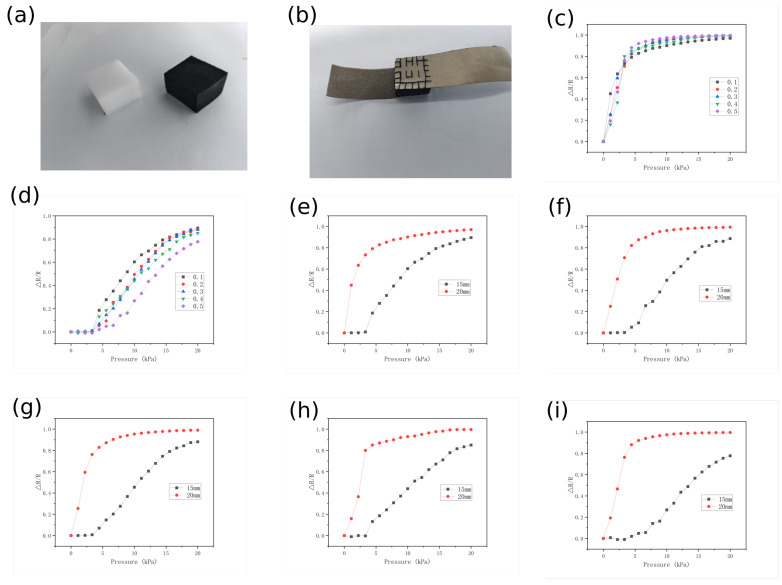
(**a**) Sponge without impregnation and impregnated sponge. (**b**) CS/CNT/MS sensor fabricated via stitching. (**c**) Resistance responses of 20 mm uncompressed sponge sensors at various concentrations. (**d**) Resistance responses of 15 mm compressed sponge sensors at various concentrations. (**e**–**i**) Resistance responses under compressed vs. uncompressed conditions at concentrations of 0.1, 0.2, 0.3, 0.4, and 0.5.

Through the comparison of the mechanical response characteristics between the prototype without pre-compression encapsulation (original thickness 20 mm) and the 15 mm pre-compressed encapsulated sensor (Figure 5), it was found that the pre-loading stitching process significantly enhanced sensitivity in low-pressure zones. Experimental data revealed that the 15 mm pre-compressed sensor exhibited exceptional signal stability (ΔR/R fluctuations < 0.01) in the low-pressure regime (<3 kPa) (Figure 5d), while the unprocessed prototype demonstrated fluctuations of 0.7–0.8 in ΔR/R (Figure 5c). The sensor, at a concentration of 0.5 (Figure 5i), exhibited a sensitivity of 0.198 ${\mathrm{kPa}}^{-1}$ in the low-pressure region (0–4 kPa) and 0.007 ${\mathrm{kPa}}^{-1}$ in the high-pressure region (4–20 kPa) when uncompressed (thickness: 20 mm). However, after compressing the sensor to a 15 mm thickness, the sensitivity in the 3–20 kPa range increased to 0.047 ${\mathrm{kPa}}^{-1}$. The resistance responses of the two processing methods at five concentrations (0.1–0.5) are shown in Figure 5e,f. It can be observed that, although the pre-treated sensors sacrificed the low-pressure (0–4 kPa) sensitivity, they expanded the linear working range to a broad 3–20 kPa compared to the unprocessed counterparts. This behavior provides critical parameters for tailoring sensor designs to specific applications.

**Figure 6:**
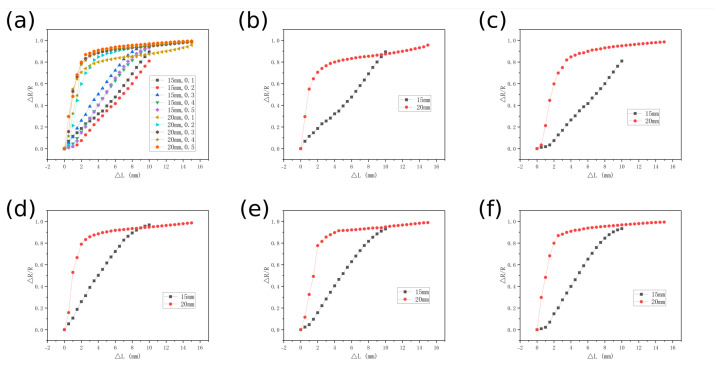
(**a**) Sensor strain test. (**b**–**f**) Strain tests of sensors with varying sewing thickness under material concentrations of 0.1, 0.2, 0.3, 0.4, and 0.5.

Through the systematic study of the pre-compression processing effects on the strain-resistance relationship of the sensor (Figure 6), it was observed that the strain-resistance response of the sensor underwent significant changes after compressed encapsulation. Figure 6b–f illustrate the resistance responses at concentrations ranging from 0.1 to 0.5; the 20 mm sensor in its original state exhibited a pronounced nonlinear strain-resistance response. This behavior can be decomposed into two distinct stages. Rapid Response Region (strain δ < 12.5%): This corresponded to the rapid closure of initial pores, during which conductive pathways exhibited exponential proliferation. This resulted in a resistance change rate of

(4) $$\frac{d(\Delta R/R)}{\Delta \delta }=0.34744.$$

Saturation Response Region (δ > 12.5%): here, impedance changes gradually plateaued with a slope of

(5) $$\frac{d(\Delta R/R)}{\Delta \delta }=0.01.$$

After pre-compressed encapsulation to 15 mm, the sensor’s response curve transitioned from nonlinear to linear. This improvement was attributed to the reduced initial resistance, R. The resistance change rate (ΔR/R) increased due to the lower R, evidenced by the slope:

(6) $$\frac{d(\Delta R/R)}{\Delta \delta }=0.09.$$

**Figure 7:**
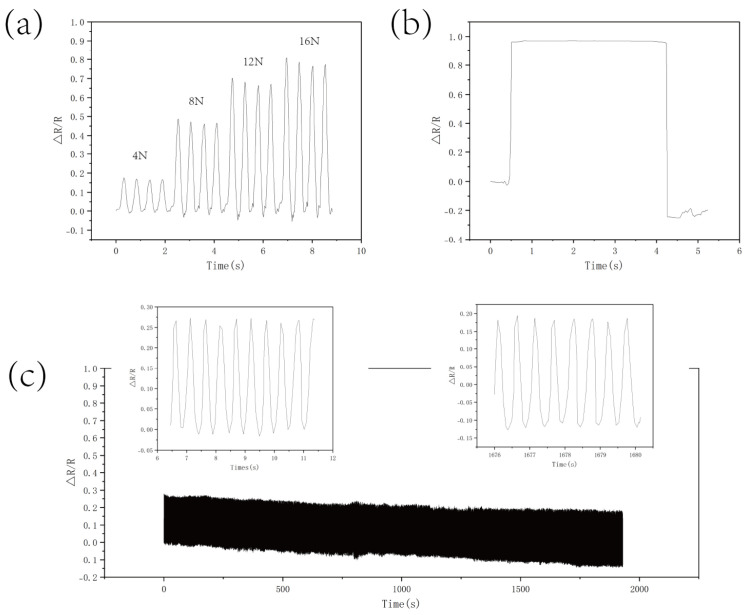
(**a**) The resistance response of the CS/CNT/MS sensor under varying pressures. (**b**) The response and recovery times of the CS/CNT/MS sensor. (**c**) The cyclic stability test of the sensor after 3500 loading/unloading cycles.

The experimental data (Figure 7a) show that the CS/CNT/MS sensor exhibited gradient resistance responses under pressures of 4 N, 8 N, 12 N, and 16 N.

Upon unloading, the resistance rapidly recovered, confirming the strict reversibility of its contact network reconfiguration. Dynamical tests (Figure 7b) indicated response/recovery times of 72 ms and 24 ms, respectively, attributed to the synergistic effect of chitosan-enhanced interfacial adhesion and nanotube conductivity mechanisms. After 3500 compression cycles (Figure 7c), the sensor’s ΔR showed relatively minor changes, indicating significant stability. This durability likely arose from chitosan anchoring CNT firmly onto the sponge skeleton, minimizing CNT detachment during compression and prolonging the sensor’s operational lifespan.

**Figure 8:**
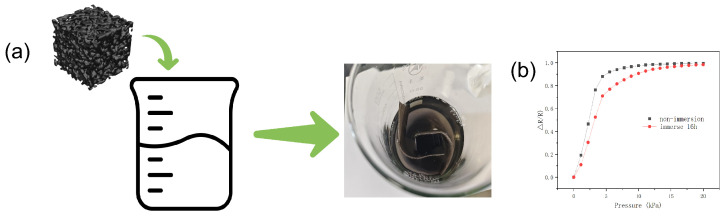
(**a**) Sensor submerged in water for 16 h; (**b**) comparison of pressure resistance response after drying.

To evaluate the waterproof performance of the sensor, a systematic experiment was conducted (Figure 8); to maximize the contrast, sensors fabricated at the 0.5 concentration were selected for experimentation.

First, the sensor’s initial resistance (R) and full-pressure-range response curves were recorded under standard dry conditions. The sensor was then fully submerged in deionized water contained in a glass beaker. After manual compression to eliminate internal bubbles and ensure 100% wetting, it was sealed and immersed in a 25 °C laboratory environment for 16 h. Post-submersion, the sensor was dried in a 60 °C forced-air oven for 8 h. Comparative tests demonstrated the following (Figure 8b): Sensitivity in the low-pressure zone (0–4 N) decreased by 19.2% compared to baseline values (attributed to reduced interfacial adhesion caused by moisture presence, with sensitivity decreasing from 0.198 ${\mathrm{kPa}}^{-1}$ to 0.160 ${\mathrm{kPa}}^{-1}$). Despite the performance degradation in the low-pressure range after 16-h immersion, the sensor maintained 90% of its initial resistance at loads exceeding 9 N, demonstrating both moisture-resistant operational capability and recovery potential upon drying.

### 3.4. Application of Sensors

**Figure 9:**
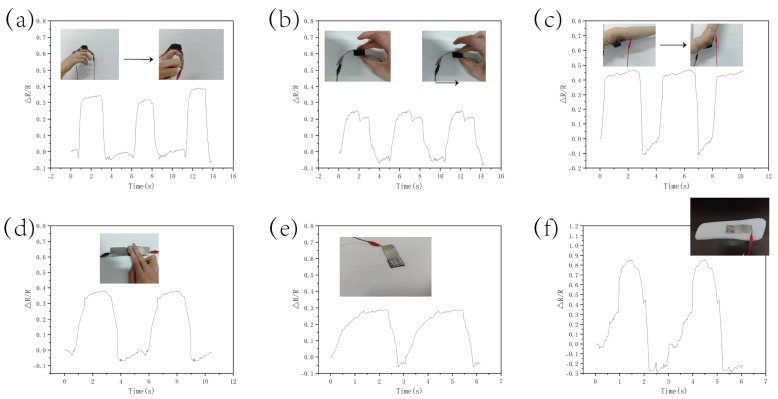
Activities monitored by the sensor during human motion tests: (**a**) finger flexion, (**b**) elbow flexion, (**c**) single-digit pinching, (**d**) dual-digit pressing, (**e**) seat cushion pressure mapping, (**f**) insole gait pressure analysis.

The CS/CNT/MS flexible pressure sensor demonstrates broad application potential in human motion monitoring due to its wide-range response characteristics. Experimental data show that in dynamic biometric joint detection (Figure 9a,b), the sensor exhibited high-resolution capture capabilities for finger flexion motions (response amplitude ΔR/R = 0.34) and elbow joint movements (ΔR/R = 0.46). When the sensor was placed on a horizontal platform (Figure 9c,d), the system could analyze the tactile mechanical features of single-finger and dual-finger interactions, enabling multidimensional human-machine interface decoding. A specially designed embedded sensor architecture achieved large-area pressure mapping through localized force transmission mechanisms: in a biomimetic seating system with an effective sensor-to-area ratio of 1:36 (Figure 9e), it enabled the real-time monitoring of postural pressure distribution (ΔR/R = 0.38); when integrated into insole devices (Figure 9f), it successfully recorded gait impact biomechanical characteristics (peak response ΔR/R = 0.85). These applications confirm the sensor’s practical value in sports biomechanics analysis, rehabilitation medical assessment, and intelligent prosthetic limb control systems.

**Figure 10:**
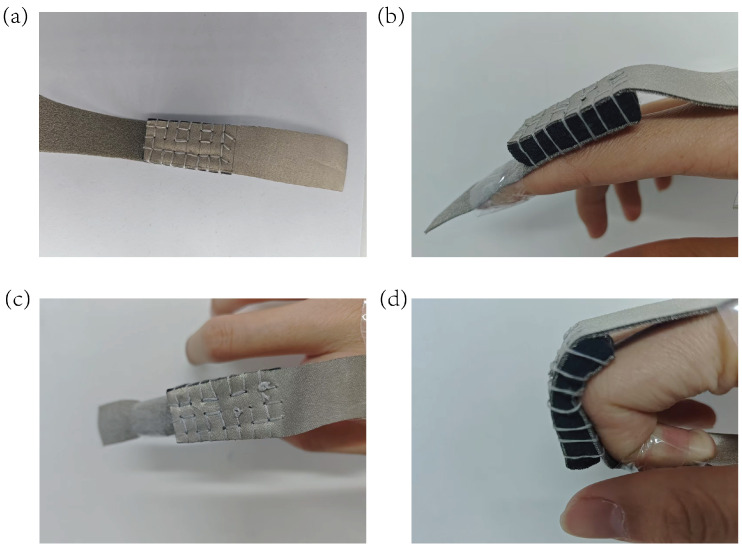
(**a**) A 20 mm × 40 mm × 10 mm rectangular−shaped sensor. (**b**) The side view of the sensor adhered to a finger. (**c**) The top view of the sensor adhered to a finger. (**d**) Sensor configuration under bending.

For adaptation to human joint bending requirements, the sensor dimensions were optimized from 30 mm × 30 mm × 15 mm to a rectangular configuration (20 mm × 40 mm × 10 mm, Figure 10a).

The redesigned structure achieved alignment with human joint kinematic characteristics (Figure 10b,c) by reducing the thickness to 10 mm and optimizing the aspect ratio (L/W = 2:1): the 20 mm width matched the phalangeal joint width of the wearer, ensuring stable adhesion; the core functional layer of the 3D conductive network (CS/CNT/MS) was fully retained (Figure 10d), enabling multi-angle bending tests without compromising the material’s intrinsic electrical properties.

**Figure 11:**
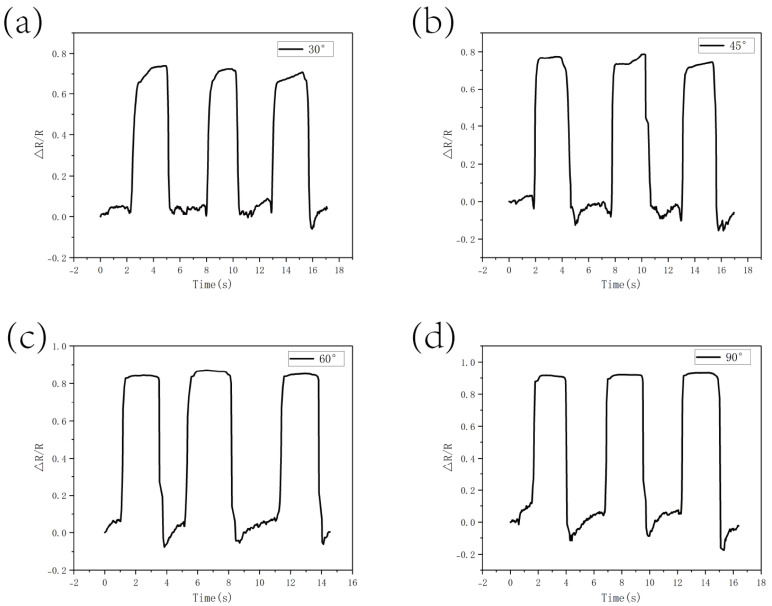
The finger bending test: (**a**–**d**) the resistance response of the sensor adhered to a finger at bending angles of 30°, 45°, 60°, and 90°.

Figure 11 demonstrates the resistance response of the CS/CNT/MS pressure sensor at bending at different angles. The experimental results indicate that the resistance change rate (ΔR/R) reached 0.7 at a small angle of 30° (Figure 11a), likely due to the rapid closure of pore structures in the melamine sponge under slight bending. This structural change triggered an exponential proliferation of conductive pathways, causing a sharp drop in resistance. As the bending angle increased to 45° (Figure 11b) and 60° (Figure 11c), the resistance change rate rose to 0.8, demonstrating the sensor’s sensitive response to angular variations. When the sensor was fully folded at 90° (Figure 11d), the resistance change rate reached 0.9. At this extreme angle, the sponge skeleton underwent significant compression, leading to partial pore closure and increased contact pressure at the CNT/CS interface. This exacerbated the reconfiguration of conductive pathways and abrupt resistance transitions. Notably, the sensor maintained continuous conductivity without exhibiting circuit breaks despite these extreme deformations.

**Figure 12:**
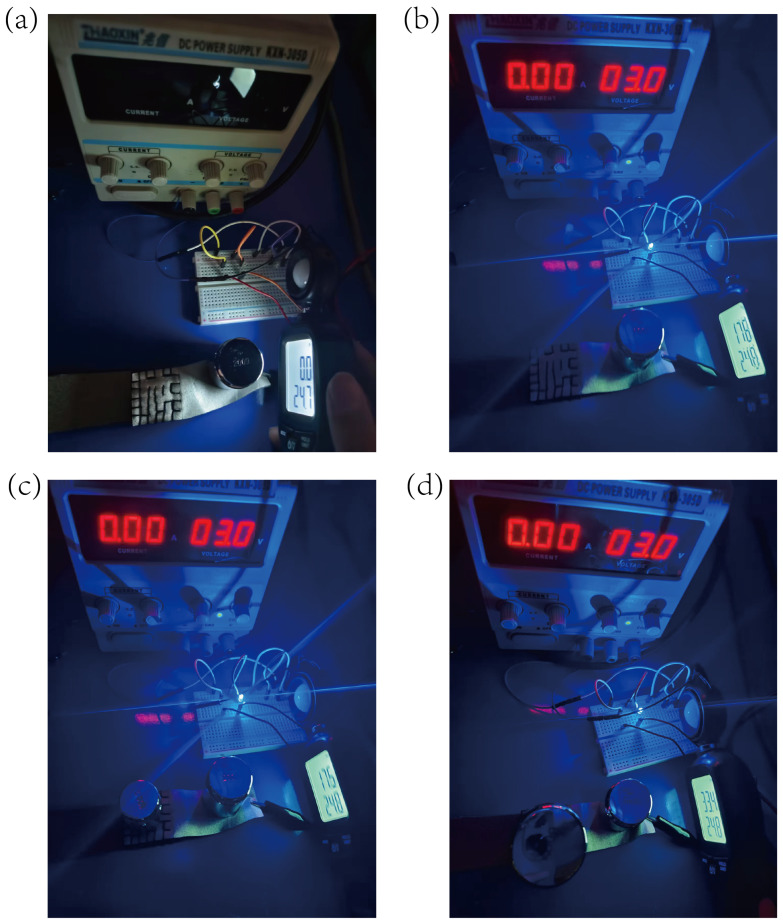
Brightness test of led: (**a**) no pressure and no voltage applied in a dark environment; (**b**) a 3 V voltage applied; (**c**) a 100 g load placed on the sponge; (**d**) a 500 g load placed on the sponge.

Through the design of an LED-visualized noise-reduction experiment (Figure 12), we validated the sensor’s ability to suppress mechanical noise: when the power was off in a dark environment, the illuminance meter read 0 lx (Figure 12a); upon applying a 3 V voltage, the LED emitted a stable luminance of 17.6 lx (Figure 12b). Under a 100 g load (2.6 kPa) (Figure 12c), the sensor’s resistance remained nearly unchanged (17.5 lx; fluctuation < 0.6%), indicating that interference below the threshold was filtered by the mechanical encapsulation structure; with a 500 g load (4.4 kPa) (Figure 12d), the LED brightness abruptly increased to 33.4 lx, an 89.8% rise from the initial value, demonstrating a significant drop in sensor resistance under effective loading.

This “on-off” response stemmed from a 3 kPa noise-suppression threshold established by the sewing encapsulation design: below the threshold, the pre-stressed balanced structure absorbed minor disturbances; above the threshold, the elastic deformation of the porous sponge skeleton drove the reconfiguration of the conductive network, enabling a high-sensitivity response, achieving <1% stability in the low-noise region and 89.8% response gain in the signal region, successfully decoupling noise from effective signal detection.

## 4. Conclusions

This study proposes a piezoresistive pressure sensor based on a sewing-based pre-compression encapsulation strategy, achieving a breakthrough integration of active noise suppression and wide-range sensing through the synergistic design of a chitosan/carbon nanotube/melamine sponge (CS/CNT/MS) composite material and a mechanical low-pass filtering mechanism. The core innovation lies in the following: (1) A pre-stress threshold system (3 kPa) was constructed via sewing-compression processing, which established dynamic equilibrium between sponge elasticity and textile forces ($F_{elastic}$ = $F_{textile}$ + $F_{external}$), forming a mechanical filtering layer in the <3 kPa interference zone to suppress environmental vibrations or accidental-touch noise ((ΔR/R) fluctuations < 0.01). (2) When pressure exceeds the threshold (3–20 kPa), the sponge skeleton’s elastic deformation overcomes the pre-stress constraint, driving linear response via pore closure and conductive pathway reconfiguration in the 3D network (sensitivity: 0.053 ${\mathrm{kPa}}^{-1}$), significantly outperforming traditional porous sensors’ nonlinear desensitization at high pressures. (3) The sensor was fabricated via a stepwise impregnation-low-temperature curing-sewing-compression process, enabling a stable conductive network via the molecular cross-linking and 3D penetration of the CS/CNT composite layer. This ensured 90% performance retention after 3500 cycles, ultra-fast response/recovery times (72 ms/24 ms), and water immersion resistance (>90% high-pressure response retention after 16-h immersion). (4) This cross-scale synergistic design of mechanical pre-filtering and electrical sensing first enables the programmable regulation of active low-frequency interference suppression and a precise linear response in the effective pressure zone, providing a novel material-structure-encapsulation synergy paradigm for noise reduction in flexible piezoresistive sensors under complex environmental conditions.

## Data Availability

No new data were created for this study.
